# Prevalence and genotype distribution of HPV6/11/16/18 infections among 180,276 outpatient females from a Women’s and Children’s Central Hospital, 2015–2021, Chengdu, China

**DOI:** 10.1038/s41598-023-48222-1

**Published:** 2023-12-14

**Authors:** Xiaoqing Wei, Junying Zhang, Youwen Mei, Qianling Dai, Xiaoli Yang, Xuemei Wang

**Affiliations:** 1 Department of Cervical Disease and Cervical Cancer Prevention and Treatment, Chengdu Women’s and Children’s Central Hospital, School of Medicine, University of Electronic Science and Technology of China, Chengdu, 611731 China; 2 Clinical Laboratory Department, Chengdu Women’s and Children’s Central Hospital, School of Medicine, University of Electronic Science and Technology of China, Chengdu, 611731 China; 3 Department of Assisted reproduction department, Chengdu Women’s and Children’s Central Hospital, School of Medicine, University of Electronic Science and Technology of China, Chengdu, 611731 China

**Keywords:** Cancer, Medical research

## Abstract

The aims of this study on human papilloma virus (HPV) 6/11/16/18 infection among females in Chengdu were to provide more targeted strategies for the prevention and treatment of cervical cancer and genital warts. In this study, the infection status of 20 genotypes was analysed by gene chip technology. The prevalence rates of HPV-6, -11, -16, and -18 infection among 180,276 cases were 0.94%, 0.57%, 3.22%, and 1.28%, respectively. The prevalence of HPV 6/11/16/18 showed a bimodal U-shaped curve with age; the first and second peak occurred among females < 20 and ≥ 60 years old, respectively. As the multiplicity of infections involving HPV6/11/16/18 increases, the infection rate decreases. The ratios of HPV16 single infection showed a yearly increase. The top five genotypes with HPV-16, -18, -6, and -11 in coinfection were HPV52/58/53/51/33, HPV 52/16/53/58/51, HPV52/16/58/51/53 and HPV16/52/58/59/18, respectively, HPV16/18/6/11 were mainly coinfected with HR-HPV. In sum, among the five vaccines available, nonavalent vaccine is more suitable for Chengdu females. For young females prioritizing vaccination is essential in the current context, while HPV screening remains an effective approach for older females. Additionally, in patients with genital warts, it is necessary to assess the presence of high-risk HPV infection and manage it appropriately in patients with genital warts.

## Introduction

Human papillomaviruses (HPVs) are non-enveloped, double-stranded circular DNA viruses of 8 kb in size that causes epithelial hyperplasia of the skin and is primarily transmitted through sexual contact or close contact and causes cervical cancer and other diseases (genital cancer, anal cancer, oropharyngeal cancers, precancers and genital warts)^1^. HPVs belong to the Papovaviridae family, and humans are the sole host. HPVs are classified into five diverse genera based on tissue tropism and subsequent host pathogenesis, which are Alphapapillomaviruses (alpha), Betapapillomaviruses (beta), Gammapapillomaviruses (Gamma), Mupapillomaviruses (mu) and Nupapillomaviruses (nu)^2^. HPV genomes are further classified into intratypic variants according to the diversity of their genome sequence^3,4^. The HPVs that infect the mucosal epithelium are classified into the genus Alphapapillomaviruses (alpha-HPVs)^5^. To date, 231 types of HPV have been identified, on the basis of the genomic sequence of L1^6^, with more than 40 types infecting the genital area^1^. Based on their carcinogenic potential, mucosal alpha-PVs are designated as high-risk and low-risk (HR-HPV and LR-HPV). Currently, at least 14 HPV genotypes have been described as high-risk (HPV16, 18, 31, 33, 35, 39, 45,51, 52, 56, 58, 59, 66, and 68)^7^, while LR-HPV includes HPV6, 11, 40, 42, 43, 44, 54, 61, 70, 72, 81, and CP6108^8^. Based on HPV activities, HPV genome is classified into three regions: the long control region (LCR) or the non-coding upstream regulatory region (URR), the late (L) region, and the early (E) region^4,5^among them, three oncogenes, E5, E6, and E7, modulate the transformation process, two regulatory proteins, E1 and E2, modulate transcription and replication, and two structural proteins, L1 and L2, compose the viral icosahedral capsid. Viral capsids have evolved to fulfil numerous roles that are critical to the establishment of viral infection^9^. HPVs infect host basal layer of the epithelium via micro-wounds^10^, and then in order to initiate viral infection, the L1 capsid protein binds to herparin sulfate proteoglycans (HSPGs) or interact with laminin-332 on the extracellular matrix Viral and capsids have evolved to fulfil numerous roles that are critical to the establishment of viral infection^11^. 60% of HPV infection in one year will be cleared spontaneously, and the great majority of infected women (more than 90%) resolve the infection spontaneously in two years^12,13^, while only a very small number of cases progress into cervical intraepithelial neoplasia (CIN), precancerous lesions and cancer. One of the reasons HPV causes cancer is that they have established molecular mechanisms to prevent cell cycle arrest and apoptosis^14^. Viral E6 and E7 oncoproteins inactivate the tumor suppressor proteins p53 and pRB, respectively, and are involved in uncontrolled cell proliferation and deliver their genetic material into the nucleus of the target cell^15,16^, Another cause of cancer is through the integration of HPV DNA into the host genome. When the E6 and E7 genes of the viral DNA remain intact and integrated into the host chromosome^17,18^, which causes additional chromosomal damages and genome destabilization in the infected cells, whereas the E6 and E7 oncogenes are extensively expressed. Approximately 98.7% of cervical cancers are attributable to HPV^19^. HR-HPV genotypes 16 and 18 are responsible for approximately 71% of cervical cancer cases^20^.

Cervical cancer is the second most common cancer in women worldwide and the third most common cause of cancer-related death worldwide. In 2020, an estimated 604127 new cases of cervical cancer and 341831 deaths worldwide were attributed to HPV infection^21^. Nearly 70% of the global burden of cervical cancer occurs in less developed countries. As the most populous developing country in the world, China bears a serious burden of cervical cancer. According to the latest statistics from the ICO/IARC HPV Information Centre in 2021, China experiences approximately 109,700 new cases and 59,100 deaths related to cervical cancer annually. The newly diagnosed cervical cancer cases in China constitute approximately 18.2% of the global incidence, while the mortality figures account for approximately 17.2% of global deaths attributed to cervical cancer^22^.

LR-HPV primarily causes genital warts and recurrent respiratory papillomatosis^1^, of which anogenital warts are more common. Ninety percent of genital warts are caused by noncarcinogenic LR-HPV genotypes 6 or/and 11, with HPV6 being the most common type of the disease, but HPV11 accounting for approximately a quarter of cases. In addition to anogenital warts, HPV types 6 and 11 are also associated with conjunctival, nasal, oral, and laryngeal warts^1^. Globally, genital warts are estimated to affect approximately 1% of the population^23^. The global incidence of genital warts in females (including both new and recurrent cases) is between 76 and 191 per 100,000 population (with a median of 120.5 per 100,000)^24^. Although genital warts are benign, they cause discomfort and affect psychological well-being. Even when addressed with physical therapy, they persist due to HPV infection and may recur upon external stimulation, which poses considerable challenges for patients and treatment.

The most effective approach for controlling cervical cancer and genital warts is prioritizing HPV vaccination. Currently, the US Food and Drug Administration (FDA) has approved three prophylactic HPV vaccines, and they are respectively Gardasil®4, a quadrivalent vaccine (6, 11, 16, 18) available in 2006, Cervarix™, a bivalent vaccine (16,18) available in 2007, and Gardasil®9, a nonavalent vaccine (6, 11, 16, 18, 31, 33, 45, 52, and 58) available in 2014^14^. As of now, the China Food and Drug Administration (CFDA) has approved a total of five vaccines, including the three mentioned above (imported bivalent, quadrivalent, and nonavalent vaccines), which were respectively marketed in mainland China in 2016, 2017, and 2018. Subsequently, in 2019 and 2022, the CFDA granted approval for two locally produced bivalent vaccines to be marketed in mainland China, namely Cecolin produced by Xiamen Wantai Canghai Biotechnology Co., Ltd. and "Wozehui" by Yunnan Walvax Biotechnology Co., Ltd. HPV vaccines have shown 98% to 100.0% protective efficacy in clinical trials against HPV type-related diseases, offering effective protection for both vaccinated and unvaccinated females^25–27^. Due to various factors, the HPV vaccine was introduced to Chinese females a decade after their global counterparts, and owing to economic constraints, the vaccine is only promoted as a national secondary vaccine. Nationally, financial constraints have resulted in less than 0.05 percent coverage of the HPV vaccine among eligible women aged 9–45 years, with women aged 9–14 years accounting for less than 5 percent of the vaccinated population^28^. Alongside vaccination, HPV DNA and cytologicals creening is crucial for alleviating the burden of cervical cancer and other HPV-related diseases. The World Health Organization's "Global Strategy to Accelerate the Elimination of Cervical Cancer" aims to have 70% of women undergo regular HPV screening by 2030, with HPV DNA testing as the preferred method for cervical cancer screening^29,30^. China conducts limited large-scale screening for female HPV DNA, with most of it taking place in hospitals. Given the high incidence of cervical cancer and related diseases in Chinese women, coupled with low HPV vaccination rates and limited HPV-DNA testing, obtaining prevalence data on HPV infection in Chinese women is vital and timely. Although infection with genotypes other than HPV6/11/16/18 can also cause genital warts and cervical cancer in women, HPV6/11/16/18 infection is the primary cause, so in-depth analysis of these four genotypes is absolutely necessary. Currently, there is a lack of large-scale research data on HPV6/11/16/18 in Chengdu or even the entire nation. The data for this study were derived from a national tertiary-grade hospital in southwest China, comprising a central institution and three branch hospitals, covering virtually the entire Chengdu urban and suburban area. This study has provided a comprehensive analysis of the situation of HPV6/11/16/18 in different age groups, infection modes, and years.

## Materials and methods

### Subjects

Patients came from Chengdu Women's and Children's Central Hospital (CWCCH) for HPV screening motivated by diverse reasons, including patient requests, diagnostic needs prescribed by doctors, and opportunistic screening by doctors. Specific reasons included health examinations, abnormal vaginal bleeding, lower genital tract inflammation, genital warts, infertility, unknown lower abdominal pain, gynaecological tumours, and urethritis. Inclusion criteria consisted of a history of sexual activity, absence from menstruation and pregnancy, while exclusion criteria included women with no sex, menstruating and pregnant women, and women who had undergone uterine surgery. For cases that were reviewed, we only considered the results of the first screening. A total of 6254 cases were excluded from the study. Finally, a total of 180,276 participants were included in this retrospective study conducted at CWCCH between January 2015 and December 2021. The mean age was 38.8 ± 9.1 years (ranging from 12 to 91 years). categorized into age groups of < 20, 20–29, 30–39, 40–49, 50–59, and ≥ 60. This study was approved by the Medical Ethics Committee of Chengdu Women’s and Children’s Central Hospital, and all methods were performed in accordance with the relevant guidelines and regulations.

### Ethics statement

This research received approval and a waiver for participant informed consent from the Medical Ethics Committee of Chengdu Women's and Children's Central Hospital due to the retrospective nature of the study, where patient identities were deliberately anonymized, rendering individual informed consent unnecessary.

### Specimen collection

A gynaecologist wipes cervical secretions with a cotton swab before sampling, places the cervical brush head on the cervix and rotates the brush head clockwise 5 times to obtain a sufficient amount of cervical epithelial cells. Then, the cervical brush head is placed into the sample tube marked with the patient's name, the cap is tightened, and the sample is quickly sent for examination.

### DNA extraction and HPV genotyping

DNA extraction and HPV genotyping were performed using an HPV genotyping test kit (Shenzhen GL Bio-Tech Co., Ltd.). The kit detected 20 types of HPV: HPV6, 11, 42, 43, 16, 18, 31,33, 35, 39, 45, 51, 52, 53.56, 58, 59, 66, 68, and 73. The process involved three steps: HPV-DNA extraction, PCR amplification and HPV-DNA hybridization. The Haema9600 (Zhuhai XZ Bio-Tech Co., Ltd.) was used for gene amplification. The amplification parameters were set as follows: ①50 °C for 2 min; ②95 °C for 10 min; ③40 cycles were performed at 95 °C for 30 s, 52 °C for 45 s and 65 °C for 30 s; and ④ 65 °C for 5 min. GL-HB-9600 (Shenzhen GL Bio-Tech Co., Ltd.) was used for DNA hybridization. After color rendering, positive detection results were indicated by clear blue dots. To ensure the reliability of each HPV test result, six probe arrays (including 3 positive anchor points, 1 negative anchor point, and 2 internal control points) were used as the internal control (IC), and HPV16 was added as a positive control. The testing doctor strictly follows the HPV interpretation rules to issue the patient's HPV test results. This study completely ruled out the impact of reproductive pathogens (BV, TV, VVC, NG, CT, etc.) other than HPV on the HPV detection results. Specific HPV extraction solution was added during the DNA extraction stage to ensure specificity. During the DNA amplification stage, HPV primers and probes were added to the reaction mixture to ensure the specific amplification of HPV fragments. The probes on the gene chip were specifically designed for HPV genotypes, ensuring no cross-reactivity between different genotypes during the DNA hybridization process.

### Statistical analysis

Microsoft's Excel 2021 was used to process and analyse the data, the chi-square test was used to compare the sample rate between the groups, SPSS 26 (SPSS Inc., Chicago, IL, USA) was used to calculate chi-square data and P values, and two-sided P values of less than 0.05 were considered statistically significant.

## Results

### Overall prevalence of HPV infection

**Figure 1:**
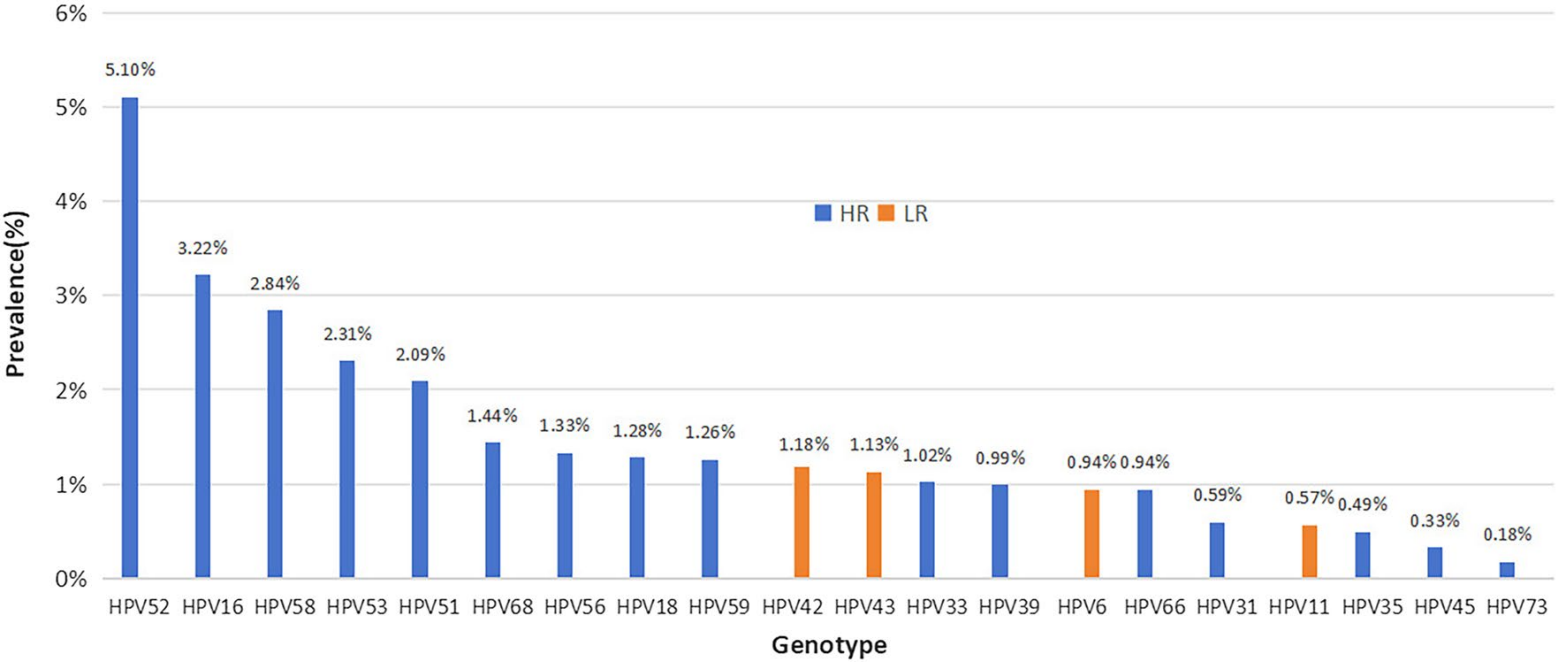
Prevalence and genotype distribution of HPV infection in 180,276 women from 2015 to 2021 (HR-HPV and LR-HPV).

The total infection rate of in 180,276 cases was 21.97% (39,615 positive cases). the 16 high-risk types were HPV-52, -16, -58, -53, -51, -68, -56, -18, -59, -33, -39, -66, -31, -35, -45 and -73 in order. The prevalence of the 20 genotypes is shown in Fig. 1, in which HPV16 and HPV18 ranked second and eighth, respectively, with infection rates of 3.22% (5801 positive cases) and 1.28% (2310 positive cases). The four low risk infection rates were HPV-42, -43, -6 and -11 in order. HPV6 and HPV11 ranked third and fourth, with infection rates of 0.94% (1699 positive cases) and 0.57% (1019 positive cases), respectively (Fig. 1, Table 1).

**Table 1:** Prevalence of HPV6/11/16/18 infections, -6 and -11 coinfection, -16 and -18 coinfection, and -6, -11, -16 and -18 coinfection in different age groups and different years and their proportions.

	Sample case(n,%)	HPV-6(n = 1699)	HPV-11(n = 1019)	HPV-16(n = 5801)	HPV-18(n = 2310)	HPV-6, -11(n = 42)	HPV-16, -18(n = 198)	HPV-6, -11, -16, -18(n = 2)	total(n,%)
Age(years)									
< 20	797(0.44)	66(8.28)	36(4.52)	60(7.53)	39(4.89)	7(0.88)	5(0.63)	1(0.13)	214(26.85)
20–29	65,432(36.3)	823(1.26)	467(0.71)	2237(3.42)	934(1.43)	23(0.04)	101(0.15)	1(0.00)	4586(7.01)
30–39	69,485(38.54)	487(0.70)	322(0.46)	2015(2.90)	782(1.13)	7(0.01)	49(0.07)	0(0.00)	3662(5.27)
40–49	29,963(16.62)	179(0.60)	97(0.32)	853(2.85)	325(1.08)	3(0.01)	27(0.09)	0(0.00)	1484(4.95)
50–59	12,002(6.66)	112(0.93)	64(0.53)	445(3.71)	179(1.49)	2(0.02)	11(0.09)	0(0.00)	813(6.77)
≥ 60	2597(14.4)	32(1.23)	33(1.27)	191(7.35)	51(1.96)	0(0.00)	5(0.19)	0(0.00)	312(12.01)
χ^2^		104.159	35.946	3.123	5.48	20.931	9.117	4.593	42.436
P		< 0.001	< 0.001	0.077	0.019	< 0.001	0.003	0.032	< 0.001
Year									
2015	20,669(11.47)	177(0.86)	123(0.60)	606(2.93)	264(1.28)	5(0.02)	30(0.15)	1(0.00)	1206(5.83)
2016	24,972(13.85)	246(0.99)	109(0.44)	732(2.93)	284(1.14)	6(0.02)	24(0.10)	0(0.00)	1401(5.61)
2017	29,944(16.61)	295(0.99)	157(0.52)	890(2.97)	421(1.41)	9(0.03)	37(0.12)	0(0.00)	1809(6.04)
2018	29,065(16.12)	336(1.16)	219(0.75)	1103(3.79)	392(1.35)	10(0.03)	27(0.09)	0(0.00)	2087(7.18)
2019	33,642(18.66)	287(0.85)	215(0.64)	1142(3.39)	394(1.17)	8(0.02)	32(0.10)	0(0.00)	2082(6.19)
2020	25,115(13.93)	188(0.75)	105(0.42)	814(3.24)	289(1.15)	1(0.00)	28(0.11)	0(0.00)	1535(6.11)
2021	16,869(9.36)	170(1.01)	91(0.54)	514(3.05)	266(1.58)	3(0.02)	20(0.12)	1(0.01)	1079(6.40)
χ^2^		1.005	0.005	7.654	0.928	1.665	0.539	0.001	10.994
P		0.316	0.944	0.006	0.335	0.197	0.463	0.975	< 0.001
Pattern of infection									
Single		825(48.56)	585(57.41	3522(60.71)	1299(56.23)				6231
Double		482(28.37)	280(27.48)	1476(25.44)	604(26.15)	11(26.19)	82(41.41)	0	2935
Triple		242(14.24)	93(9.13)	524(9.03)	230(9.96)	16(38.10)	54(27.27)	0	1159
Quad		90(5.30)	40(3.93)	175(3.02)	119(5.15)	6(14.29)	38(19.19)	1(50.00)	469
Five		38(2.24)	14(1.37)	70(1.21)	37(1.60)	5(11.90)	14(7.07)	0	178
Six		9(0.53)	4(0.39)	19(0.33)	13(0.56)	1(2.38)	7(3.54)	0	53
Seven		10(0.59)	3(0.29)	10(0.17)	4(0.17)	3(7.14)	1(0.51)	1(50.00)	32
Eight		2(0.12)	0(0)	4(0.07)	3(0.13)	0	1(0.51)	0	10
Nine		1(0.06)	0(0)	1(0.02)	1(0.04)	0	1(0.51)	0	4

**Table 2:** Prevalence of HPV6/11/16/18 infection patterns in different age groups and in every year.

	HPV6 (n,%)					HPV11 (n,%)				
	Single	Double	Triple	Quad+	Total	Single	Double	Triple	Quad+	Total
Age (years)										
< *20(n* = *797)*	15 (1.88)	18 (2.26)	14 (1.76)	19 (2.38)	66 (8.28)	16 (2.01)	7 (0.88)	5 (0.63)	8 (1.0)	36 (4.52)
*20–29 (n* = *65,432)*	387 (0.59)	227 (0.35)	126 (0.19)	83 (0.13)	823 (1.26)	261 (0.40)	128 (0.20)	51 (0.08)	27 (0.04)	467 (0.71)
*30–39(n* = *69,485)*	264 (0.38)	146 (0.21)	50 (0.07)	27 (0.04)	487 (0.70)	197 (0.28)	91 (0.13)	22 (0.03)	12 (0.02)	322 (0.46)
*40–49(n* = *29,963)*	99 (0.33)	52 (0.17)	22 (0.07)	6 (0.02)	179 (0.60)	57 (0.19)	27 (0.09)	8 (0.03)	5 (0.02)	97 (0.32)
*50–59(n* = *12,002)*	48 (0.40)	32 (0.27)	22 (0.18)	10 (0.08)	112 (0.93)	37 (0.31)	19 (0.16)	4 (0.03)	4 (0.03)	64 (0.53)
≥ *60(*= *2597)*	12 (0.46)	7 (0.27)	8 (0.31)	5 (0.19)	32 (1.24)	17 (0.65)	8 (0.31)	3 (0.12)	5 (0.19)	33 (1.28)
*χ*^2^	33.881	27.661	14.236	40.502	104.159	16.055	7.628	12.651	3.041	35.946
*P*	< 0.001	< 0.001	< 0.001	< 0.001	< 0.001	< 0.001	0.006	< 0.001	0.081	< 0.001
Year										
2015	69 (38.98)	54 (30.51)	29 (16.38)	25 (14.12)	177	65 (52.85)	34 (27.64)	13 (10.57)	11 (8.94)	123
2016	117 (47.56)	64 (26.02)	38 (15.45)	27 (10.98)	246	63 (57.80)	30 (27.52)	7 (6.42)	9 (8.26)	109
2017	141 (47.80)	90 (30.51)	44 (14.92)	20 (6.78)	295	94 (59.87)	42 (26.75)	11 (7.01)	10 (6.37)	157
2018	160 (47.62)	107 (31.85)	45 (13.39)	24 (7.14)	336	118 (53.88)	65 (29.68)	25 (11.42)	11 (5.02)	219
2019	141 (49.13)	81 (28.22)	40 (13.94)	25 (8.71)	287	128 (59.53)	55 (25.58)	22 (10.23)	10 (4.65)	215
2020	99 (52.66)	42 (22.34)	26 (13.83)	21 (11.17)	188	52 (49.52)	38 (36.19)	9 (8.57)	6 (5.71)	105
2021	98 (57.65)	44 (25.88)	20 (11.76)	8 (4.71)	170	65 (71.43)	16 (17.58)	6 (6.59)	4 (4.40)	91

	HPV16 (n,%)					HPV18 (n,%)				
	Single	Double	Triple	Quad+	Total	Single	Double	Triple	Quad+	Total
Age (years)										
<*20(n = 797)*	14 (1.76)	15 (1.88)	14 (1.76)	17 (2.13)	60 (7.53)	10 (1.25)	13 (1.63)	6 (0.75)	10 (1.25)	39 (4.89)
*20–29 (n = 65,432)*	1207 (1.84)	633 (0.97)	256 (0.39)	141 (0.22)	2237 (3.42)	475 (0.73)	257 (0.39)	116 (0.18)	86 (0.13)	934 (1.43)
*30–39(n = 69,485)*	1351 (1.94)	469 (0.67)	142 (0.20)	53 (0.08)	2015 (2.90)	488 (0.70)	194 (0.28)	58 (0.08)	42 (0.06)	782 (1.13)
*40–49(n = 29,963)*	577 (1.93)	207 (0.69)	51 (0.17)	18 (0.06)	853 (2.85)	206 (0.69))	79 (0.26)	28 (0.09)	12 (0.04)	325 (1.08)
*50–59(n = 12,002)*	285 (2.37)	99 (0.82)	31 (0.26)	30 (0.25)	445 (3.71)	98 (0.82)	45 (0.37)	19 (0.16)	17 (0.14)	179 (1.49)
≥ *60(= 2597)*	88 (3.39)	53 (2.04)	30 (1.16)	20 (0.77)	191 (7.42)	22 (0.85)	16 (0.62)	3 (0.12)	10 (0.39)	51 (1.98)
*χ*^2^	23.794	2.264	8.767	3.171	3.123	0.099	4.123	9.898	3.701	5.48
*P*	< 0.001	0.132	0.003	0.075	0.077	0.753	0.042	0.002	0.054	0.019
Year										
2015	329 (54.29)	181 (29.87)	54 (8.91)	42 (6.93)	606	152 (57.58)	61 (23.11)	26 (9.85)	25 (9.47)	264
2016	423 (57.79)	213 (29.10)	55 (7.51)	41 (5.60)	732	161 (56.69)	68 (23.94)	25 (8.80)	30 (10.56)	284
2017	536 (54.64)	219 (22.32)	91 (9.28)	44 (4.49)	981	241 (57.24)	101 (23.99)	49 (11.64)	30 (7.13)	421
2018	665 (60.29)	280 (25.39)	116 (10.52)	42 (3.81)	1103	232 (59.18)	104 (26.53)	40 (10.20)	16 (4.08)	392
2019	699 (61.21)	285 (24.96)	106 (9.28)	52 (4.55)	1142	203 (51.52)	117 (29.70)	40 (10.15)	34 (8.63)	394
2020	522 (64.13)	174 (21.38)	69 (8.48)	49 (6.02)	814	146 (50.52)	88 (30.45)	27 (9.34)	28 (9.69)	289
2021	348 (67.70)	124 (24.12)	33 (6.42)	9 (1.75)	514	164 (61.65)	65 (24.44)	23 (8.65)	14 (5.26)	266

**Table 3:** Prevalence of HPV6/11/16/18 infections at different ages from 2015 to 2021.

Year	Age (n,%)						χ^2^	P
	< 20 (n = 797)	20–29(n = 65,432)	30–39(n = 69,485)	40–49(n = 29,963)	50–59(n = 12,002)	** ≥ **60(n = 2597)		
HPV6								
2015	8 (1.00)	104 (0.16)	36 (0.05)	18 (0.06)	10 (0.08)	1 (0.04)	32.724	< 0.001
2016	13 (1.63)	125 (0.19)	58 (0.08)	30 (0.10)	16 (0.13)	4 (0.15)	21.061	< 0.001
2017	14 (1.76)	131 (0.20)	85 (0.12)	39 (0.13)	21 (0.17)	5 (0.19)	10.659	< 0.001
2018	11 (1.38)	169 (0.26)	100 (0.14)	31 (0.10)	19 (0.16)	6 (0.23)	28.078	< 0.001
2019	10 (1.25)	144 (0.22)	87 (0.13)	23 (0.08)	16 (0.13)	7 (0.27)	23.483	< 0.001
2020	8 (1.00)	82 (0.13)	61 (0.09)	22 (0.07)	12 (0.10)	3 (0.12)	7.952	0.005
2021	2 (0.25)	68 (0.10)	60 (0.09)	16 (0.05)	18 (0.15)	6 (0.23)	0.055	0.814
HPV11								
2015	1(0.13)	66(0.10)	38(0.05)	12(0.04)	5(0.04)	1(0.04)	13.412	< 0.001
2016	6(0.75)	58(0.09)	28(0.04)	10(0.03)	4(0.03)	3(0.12)	15.449	< 0.001
2017	5(0.63)	64(0.10)	54(0.08)	20(0.07)	10(0.08)	4(0.15)	2.052	0.152
2018	6(0.75)	101(0.15)	69(0.10)	25(0.08)	12(0.10)	6(0.23)	7.611	0.006
2019	11(1.38)	101(0.15)	60(0.09)	16(0.05)	19(0.16)	8(0.31)	7.411	0.006
2020	2(0.25)	43(0.07)	40(0.06)	5(0.02)	8(0.07)	7(0.27)	0.038	0.846
2021	5(0.63)	34(0.05)	33(0.05)	9(0.03)	6(0.05)	4(0.15)	0.812	0.368
HPV16								
2015	7(0.88)	269(0.41)	171(0.25)	117(0.39)	26(0.22)	16(0.62)	35.936	< 0.001
2016	8(0.88)	297(0.45)	238(0.34)	120(0.40)	53(0.44)	16(0.62)	0.452	0.502
2017	10(1.25)	315(0.48)	306(0.44)	159(0.53)	67(0.56)	33(1.27)	8.26	0.004
2018	11(1.38)	454(0.69)	379(0.55)	164(0.55)	65(0.54)	30(1.16)	3.459	0.063
2019	14(1.76)	420(0.64)	431(0.62)	133(0.44)	108(0.90)	36(1.39)	1.68	0.195
2020	7(0.88)	319(0.49)	288(0.41)	87(0.29)	75(0.62)	38(1.46)	2.288	0.13
2021	3(0.38)	163(0.25)	202(0.29)	73(0.24)	51(0.42)	22(0.85)	15.685	< 0.001
HPV18								
2015	3(0.38)	120(0.18)	83(0.12)	46(0.15)	11(0.09)	1(0.04)	9.004	0.003
2016	9(1.13)	112(0.17)	106(0.15)	43(0.14)	10(0.08)	4(0.15)	8.166	0.004
2017	5(0.63)	166(0.25)	143(0.21)	74(0.25)	20(0.17)	13(0.50)	0.289	0.591
2018	4(0.50)	171(0.26)	120(0.17)	54(0.18)	36(0.30)	7(0.27)	1.146	0.284
2019	10(1.25)	154(0.24)	133(0.19)	47(0.16)	37(0.31)	13(0.50)	0.017	0.897
2020	3(0.38)	113(0.17)	100(0.14)	34(0.11)	31(0.26)	8(0.31)	0.328	0.567
2021	5(0.63)	98(0.15)	97(0.14)	27(0.09)	34(0.28)	5(0.19)	0.382	0.537

### Age-specific prevalence of HPV6/11/16/18 infection

The 30–39 years group made up the largest proportion of screened participants, accounting for 38.54% of the total population, yet the < 20 and ≥ 60 age groups made up fewer proportions, accounting for 0.44% and 14.4%, respectively (Table 1).

**Figure 2:**
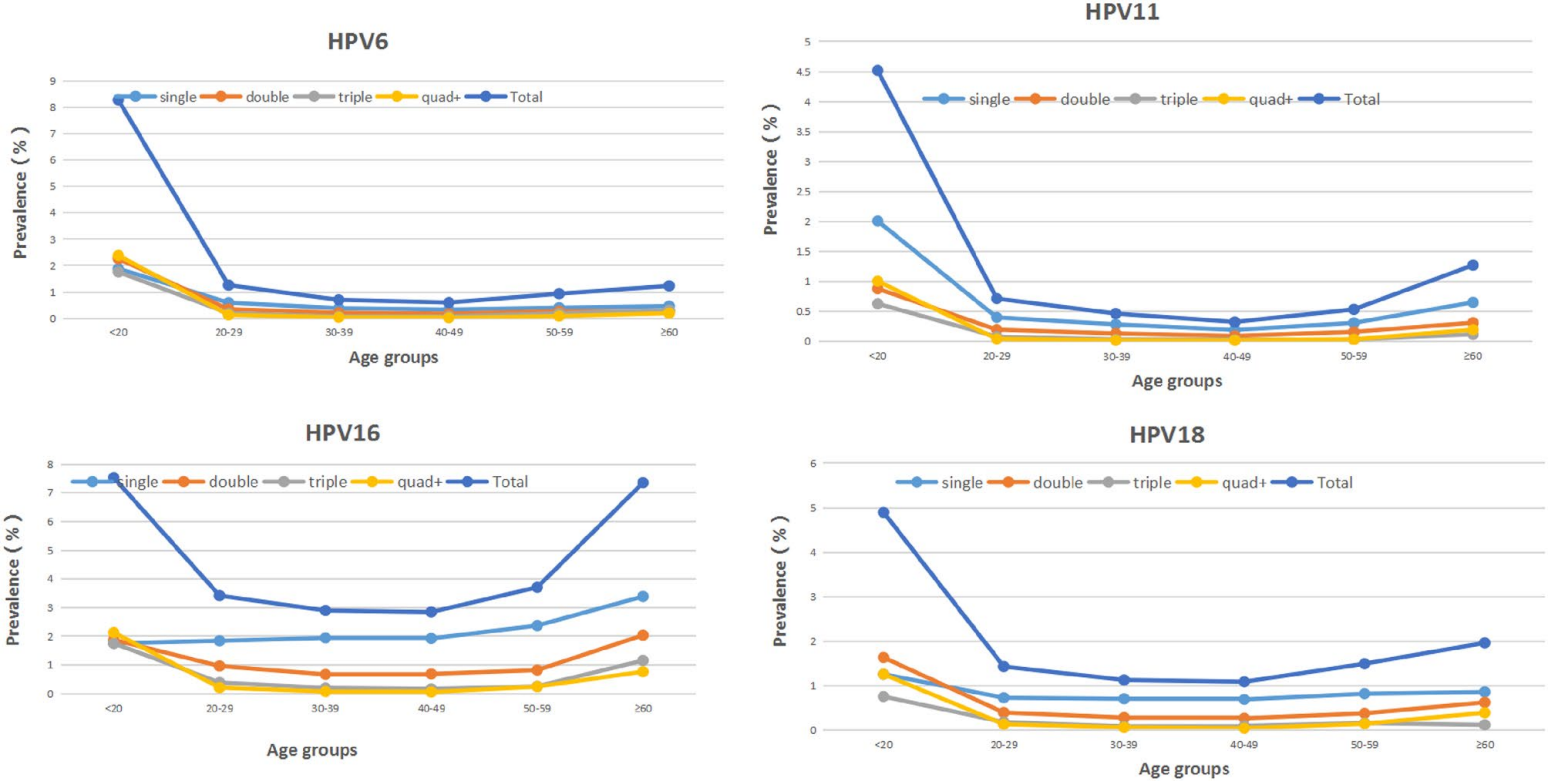
Prevalence of HPV6/11/16/18 infection patterns at different ages from 2015 to 2021.

**Figure 3:**
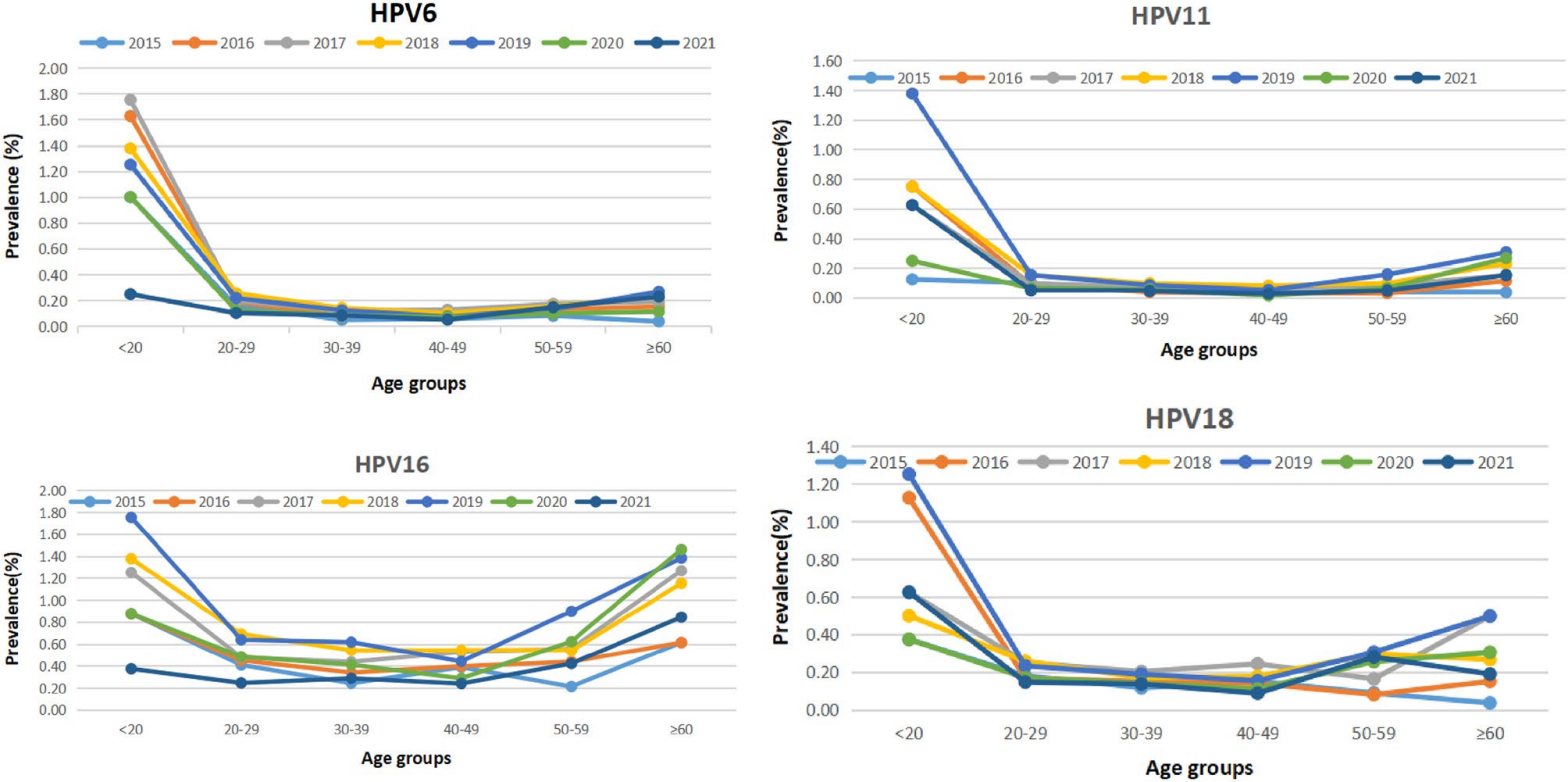
Prevalence of HPV6/11/16/18 infections in different age groups from 2015 to 2021.

The infection curves of HPV-6, -11, -16 and -18 showed a bimodal U-shaped distribution with age, as did the coinfective genotypes of -16 and -18 (Tables 1, 2, 3 and Figs. 2, 3). The first peak occurred in females < 20 years old (at this age group, the prevalence of HPV-6, -11, -16 and -18 were 8.28% and 4.52%, 7.53% and 4.89%, respectively), sharply declining after the first peak and maintaining a plateau during middle age (40–49 years), followed by a slight increase with age, reaching a small second peak in the ≥ 60 age group (at this age group, the prevalence of HPV-6, -11, -16 and -18 were 1.23% and 1.27%, 7.35% and 1.96%, respectively) (Table 1). The prevalence of HPV6/11/18 infections, HPV-6 and -11 coinfection, HPV-16 and -18 coinfection, and HPV-6, -11, -16 and -18 coinfection with age was significantly different (P < 0.05) (Table 1).

### Type-specific prevalence of HPV6/11/16/18 infection

**Figure 4:**
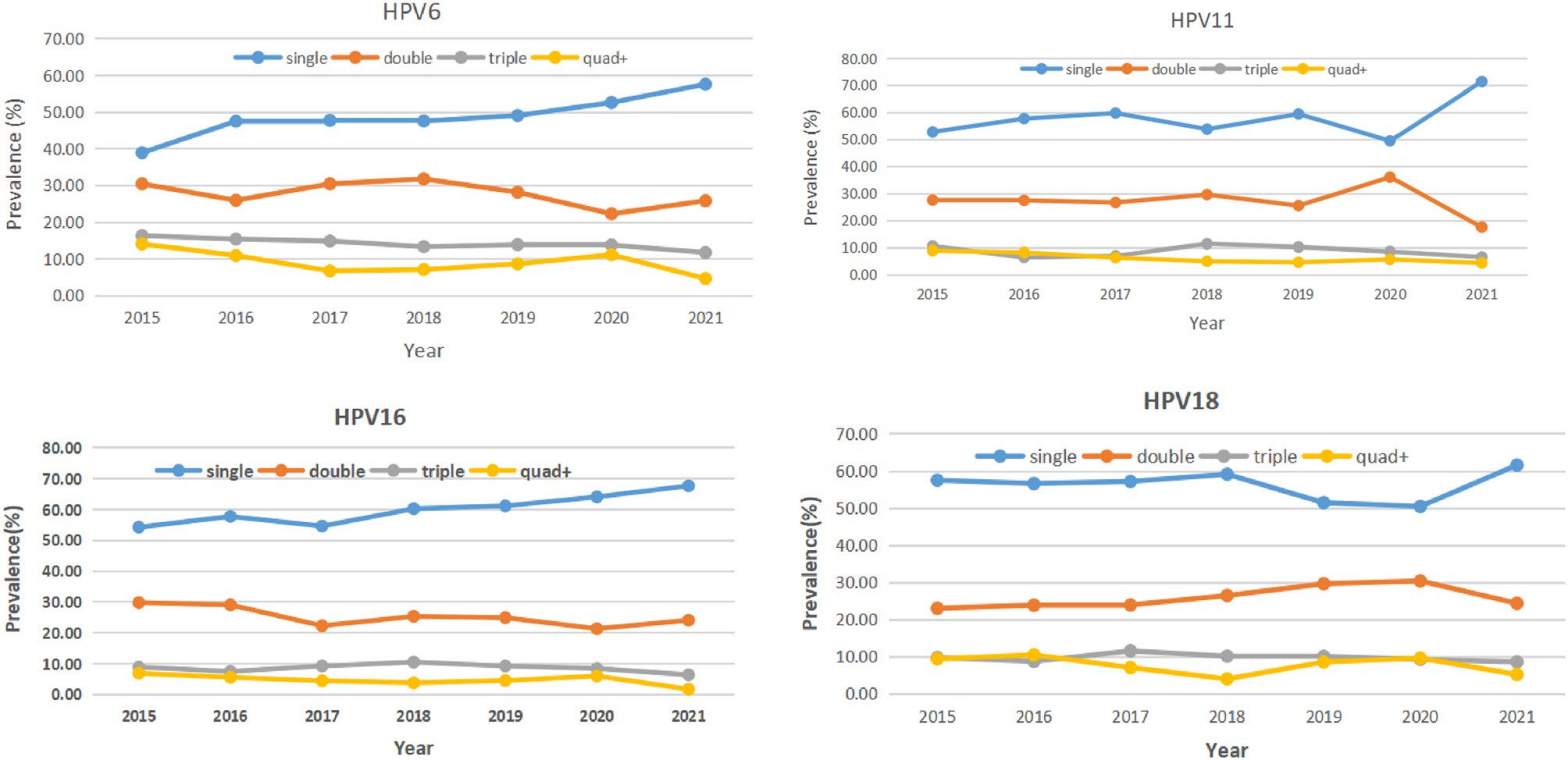
Proportion of HPV6/11/16/18 genotype-positive infection patterns for each year from 2015–2021.

**Figure 5:**
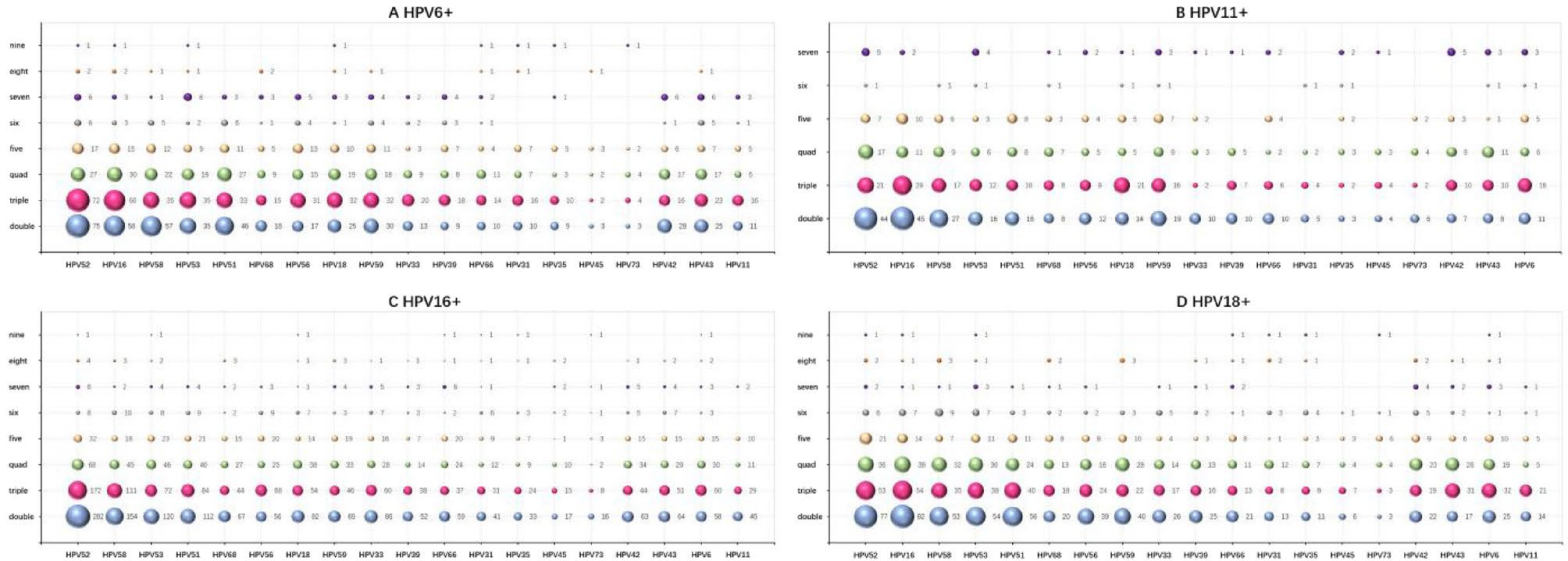
Multiple infection patterns of any one genotype of HPV6/11/16/18 with 19 other genotypes in 2015–2021.

The single infection ratios of HPV-6, -11, -16 and -18 were 48.56%, 57.41%, 60.71% and 56.23%, respectively. The double infection ratios were 28.37%, 27.48%, 25.44% and 26.15%, respectively. The triple infection ratios were 14.24%, 9.13%, 9.03% and 9.96%, respectively (Table 1). Single and multiple infections are almost equal, and double infections account for approximately half of single infections. With the increase in the patterns of multiple infections, the prevalence of multiple infections decreased gradually. This trend was observed almost every year and in almost every age group from 2015 to 2021 (Tables 1, 2 and Figs. 2, 4, 5). From 2015 to 2021, the ratios of single HPV16 infections showed a yearly increasing trend (Table 2 and Figs. 2, 4).

In the four genotypes of HPV6/11/16/18, whether HR-HPV or LR-HPV, they all present a variety of coinfection forms with other 19 genotypes, and are mainly coinfected with HR-HPV. The top ten genotypes of multiple infections with HPV-6, -11, -16, and -18 were HPV-52, -16, -58, -53, -51, -18, -59, -56, -6, and -43 (Table 4, Fig. 5). The top five genotypes with HPV-6 multiple infections were HPV-52, -16, -58, -51, and -53 in order. The top five genotypes with HPV11 multiple infection were HPV-16, -52, -58, -59, and -18 in order. The top five genotypes with HPV16 multiple infection were HPV-52, -58, -53, -51, and -33 in order. The top five genotypes with HPV18 multiple infections were HPV-52, -16, -53, -58, and -51 in order (Table 4, Fig. 5).

**Table 4:** Top 10 genotypes in multiple infections with HPV6/11/16/18 in 2015–2021.

Genotype	Multiple infections cases	No.1(cases)	No.2(cases)	No.3(cases)	No.4(cases)	No.5(cases)	No.6(cases)	No.7(cases)	No.8(cases)	No.9(cases)	No.10(cases)
HPV-6	874	HPV52(206)	HPV16(172)	HPV58(133)	HPV51(126)	HPV53(110)	HPV59(100)	HPV18(92)	HPV56(85)	HPV43(84)	HPV42(74)
HPV-11	445	HPV16(97)	HPV52(95)	HPV58(60)	HPV59(54)	HPV18(47)	HPV51(42)*	HPV53(42)*	HPV6(42)*	HPV43(34)	HPV42(33)
HPV-16	2279	HPV52(575)	HPV58(343)	HPV53(276)	HPV51(270)	HPV33(203)	HPV18(198)	HPV56(181)	HPV59(177)	HPV6(172)*	HPV43(172)*
HPV-18	1011	HPV52(198)*	HPV16(198)*	HPV53(145)	HPV58(140)	HPV51(135)	HPV59(106)	HPV56(92)*	HPV6(92)*	HPV43(85)	HPV42(84)
HPV-6, -11	42	HPV52(9)*	HPV16(9)*	HPV59(9)*	HPV18(6)	HPV42(5) *	HPV43(5) *	HPV51(4) *	HPV56(4) *	HPV58(4) *	HPV53(2)
HPV-16, -18	198	HPV52(32)	HPV58(21)	HPV51(20)	HPV53(16)	HPV42(15)	HPV43(13)	HPV33(12) *	HPV56(12) *	HPV59(11) *	HPV11(11) *
HPV-6, -11,-16, -18	2	HPV52(1) *	HPV42(1) *	HPV43(1)*							

*This means that the number of adjacent genotypes is the same, and their sequences are the same.

### Year-specific prevalence of HPV6/11/16/18 infection

From 2015 to 2021, HPV16 consistently exhibited the highest infection rate (Table 1). With the exception of the five age distribution curves of HPV-6, -11, and -18 in 2015 and HPV-18 in 2018 and 2021 (the second peak was not pronounced), the age distribution curves of HPV-6, -11, -16, and -18 from 2015 to 2021 displayed a U-shaped . From 2015 to 2020, the annual infection rates of HPV6 significantly differed with age (P < 0.05). The infection rates of HPV11 were significantly different with age in 2015, 2016, 2018, and 2019 (P < 0.05). The age-related infection rates of HPV16 were significantly different in 2015, 2017, and 2021 (P < 0.05), and the age-related infection rates of HPV18 showed significant differences in 2015 and 2016 (P < 0.05) (Table 3, Fig. 3).

## Discussion

To date, vaccination and HPV screening for women constitute two major strategies in the prevention of cervical cancer and genital warts. The focus of HPV vaccine research lies within the L region^31^ and the currently available HPV vaccines are designed using a combination of multiple subtypes of L1 virus-like particles (L1-VLPs), and the L1 spontaneously formed VLPs are highly immunogenic and produce high titers of neutralizing antibodies to prevent HPV infection^14,32^. VLPs mimic the organization and conformation of authentic native viruses but lack the viral genome, inducing an immune response without causing disease^33^. In contrast, attenuated or inactivated viruses may pose risks of incomplete attenuation or reversion to virulence, raising safety concerns. As a result, current HPV vaccines are safer than attenuated or inactivated viruses. A decade of HPV vaccination practice has demonstrated that HPV vaccine can reduce HPV infection rates, genital warts, low-grade and high-grade cervical intraepithelial neoplasia (CIN), and CIN2+^27^, moreover it has a strong group effect or indirect protection against unvaccinated females^26^. In terms of HPV screening, HPV DNA has a high sensitivity and negative predictive value (fewer high-grade lesions in the subsequent third year of screening) and is an effective screening method^34^.

In this study, the prevalence rates of HPV-16 and -18 infections in Chengdu were 3.22% and 1.28%, respectively. These rates were significantly higher than those of the less developed regions of Guangxi^35^ (2.7% and 1.11%, respectively) and the more developed regions of Shanghai^36^ (2.34% and 1.0%, respectively) but lower than those of the economically comparable Tianjin^37^ (5.36% and 1.57%, respectively). The prevalence rates of HPV-6 and -11 infections in Chengdu (0.94% and 0.57%, respectively) were lower than those in Shanghai^36^ (1.29% and 0.81%, respectively) and Guangxi^35^ (1.31% and 0.82%, respectively) but higher than those in Tianjin^37^ (0.30% and 0.51%, respectively). Disparities in HPV infection rates across different regions may be influenced by factors such as local economic conditions, awareness of prevention, lifestyle habits, and HPV detection methods. However, regardless, given the crucial roles of HPV 16/18 and HPV 6/11 in cervical cancer and genital warts, the aforementioned findings underscore the imperative need to enhance HPV vaccine coverage in the Chengdu region.

In terms of infection patterns, the ratios of HPV-6, -11, -16 and -18 single infection were 48.56%, 57.41%, 60.71% and 56.23%, respectively, which indicates that single infection is the primary mode of HPV infection, followed by double infection. As the multiplicity of coinfection increased, the prevalence of multiple infections gradually decreased. The occurrence of multiple infections is associated with having multiple sexual partners. The potential impact of multiple infections on cervical cancer risk remains debatable^38,39^. In this study, the top five genotypes in 180,276 cases were HPV-52,-16,-58,-53,-51, and the top five genotypes with HPV16 and HPV18 coinfection were HPV52, -58, -53, -51, -33 and HPV52, -16, -53, -58, -51, respectively, while the top five genotypes with HPV6 and HPV11 coinfection were HPV-52, -16, -58, -51, -53 and HPV-16, -52, -58, -59, -18, respectively. Thus it can be seen that HPV-52 and -58 are the top two genotypes among females in Chengdu. However, currently, among the five available HPV vaccines in the China mainland, only the nonavalent vaccine covers these two genotypes, so the nonavalent vaccine is more suitable for Chengdu females. These findings of this large-scale epidemiological investigation based on HPV genotyping screening hold significance for the prevention and control of diseases such as cervical cancer and genital warts in Chengdu. Additionally, they provide valuable insights for the future design of HPV vaccines.

HPV16, as a highly carcinogenic genotype, was found to have the highest infection rates in HPV6/11/16/18. The single infection positivity rate of HPV16 showed a continuous increase yearly, and studies^40^ have indicated that the proportion of high-grade cervical lesions among HPV16 single infections is the highest. In response to the increasing trend of HPV16 single infections, a stronger push for vaccines covering HPV 16 is warranted. While the infection rate of HPV18 is lower than that of HPV16, its coinfections with HPV52, -58, -53, -51, and -33 genotypes in multiple infections probably enhance its carcinogenic potential.

In this study, the coinfection of HPV6 and HPV11 was very low (42 cases), the single infection of HPV6 and HPV11 accounted for the majority (48.56% and 57.41%, respectively), and most of them had multiple coinfections with HR-HPV. In cases of multiple infections involving HPV 6, the most frequently cooccurring genotype is HPV-52, -16, -58, -51, and -53, whereas for HPV 11, the most commonly cooccurring genotype is HPV-16, -52, 58, -59, and -18. It can be seen that low-risk HPV6 and HPV11 are the most common co-infected LR-HPV with HR-HPV. Studies^41^ have pointed out that the most common HPV genotypes in genital warts in China are HPV-6, HPV-11 and HPV-16. In this study, HPV16 ranked second in HPV6 multiple infections (172 coinfections), and HPV16 ranked first in HPV11 multiple infections (97 coinfections), which also explains why HPV16 is the most frequent HR-HPV in genital warts. Moreover, multiple infections of HPV6/11 with HR-HPV may be the cause of genital warts appearing in LSIL, HSIL and tumour progression^42^; therefore, in patients with genital warts, it is necessary to test whether there is an HR-HPV infection and to address it appropriately.

In this study, the prevalence of HPV6/11/16/18 was bimodal with age, with young women (< 20 years old) having the highest prevalence, old women (≥ 60 years old) having higher prevalence, and middle-aged women (20–49 years old) having a modest decline in prevalence. In fact, HPV infection is bimodal with age and is very common in many regions, and almost all young women have the first peak, with the second peak occurring at different ages^35–37,43,44^. The first peak of HPV infection rates in young women can be up to 80% in some populations^45^, with most women suffering from transient infections that clear rapidly^46^. The highest infection rate among young women (< 20 years old) may be related to less mature immune protection and/or multiple sexual partners in this age bracket^47^ but also to the fact that HPV screening is performed only if the patient has a sexual history and associated symptoms (e.g., genital warts) or if the patient requests it, which results in a relatively high detection rate. In the face of the highest HPV6/11/16/18 infection rate among young girls, it is urgent to strengthen HPV vaccination among women who have not had sex before in Chengdu. After this first peak, the prevalence of infection gradually declined to a plateau in middle-aged women (20–49 years old), which is probably due to increased autoimmune function and stable sexual partners. For the second minor peak of infection, the higher rate of infection in old women (≥ 50 years old) may be due to immunosenescence, changes in sexual behaviour and sexual partner (both for male and female), and reappearance of past (latent) infections^44,48–51^. Some scholars^52^ point out that the geographic difference in this second peak may be partially explained by indirect indicators of menopausal hormonal patterns, such as body mass index (BMI) and ethnicity. Since young women (age ≤ 20 years) are HPV-susceptible because of high HPV infection rates, vaccination of women before sexual debut is the most cost-effective strategy to prevent cervical cancer. Data^53,54^ showed that the earlier young women were inoculated with the HPV vaccine, the higher the antibody titre and the better the protective effect of the vaccine. Studies^55^ showed that almost everywhere, most men and women started having sex in their late teens (ages 15–19), and most began having sex at 15. The crude median sexual debut age for the youngest age group was 17 years in China^56^. Considering Chinese cultural traditions and social factors such as nine-year compulsory education, Chinese scholars recommend that junior middle school girls (aged 13 to 15) to be the first group to be vaccinated. In mainland China, there is an ongoing initiative to gradually introduce free HPV vaccination services or provide subsidized HPV vaccine inoculation. Currently, 19 provinces, municipalities, or autonomous regions across China offer free HPV vaccine administration. As of now, locations within Sichuan Province where free HPV vaccination can be arranged for those junior middle school girls (aged 13 to 15) include the entire city of Chengdu, as well as Wenchuan County in Aba and the city of Barkam, the latter two being economically disadvantaged areas. Similarly, HPV vaccination is also necessary in women from less than the age of 20 to 45, and even in women already infected with HPV, data^54^ show that the immune response to the HPV vaccine appears to prevent reinfection or reactivation of disease with vaccine HPV type. Moreover, HPV 16 and 18 vaccines were well tolerated and highly efficacious against HPV 16 and 18-associated high-grade genital lesions and persistent infection^57^.

For the second minor peak of HPV infection, HPV and cytological screening is especially important in middle-aged and older women, as screening not only reduces the burden of precancerous lesions and related persistent HPV infections, but removal of lesions may have a direct antigen-presenting effect that could protect against subsequent HPV infections^58^. Given that HPV 16 or 18 infections have the highest risk for CIN 3 and occult cervical cancer, 2019 ASCCP^59^ specifically emphasized that additional evaluation (e.g., colposcopy with biopsy) is necessary even when cytology results are negative if HPV 16 or 18 testing is positive and that additional laboratory testing of the same sample is not feasible, the patient should proceed directly to colposcopy.

In sum, vaccination is of extreme importance in the current situation. The WHO global strategy to accelerate the elimination of cervical cancer as a public health problem highlights 90% vaccination coverage for girls younger than 15 years old by 2030 as one of the three targets^60^. For middle-aged and older women, regular HPV screening is a better strategy to prevent cervical cancer and precancerous lesions.

The study possesses several limitations. First, it lacked cell or pathological data from positive cases, rendering comparative statistics unfeasible. Second, female vaccination rates were not taken into account, making it impossible to calculate the specific impact of the vaccine on HPV infection rates. Third, the study's subjects comprised solely patients from hospitals within a 6-year timeframe, which does not represent the typical population of Chengdu. Therefore, these findings are exploratory in nature and merit further investigation. Fourth, only four genotypes were involved in this study, obviously, some other HR-HPVs and LR-HPVs are involved in malignant disorders, especially cervical cancer and other genital cancers, However, due to the need for detailed and in-depth study of the four genes, few other genotypes are taken in account, which need to be further presented in future studies.

## Conclusions

In conclusion, the infection rate of HPV6/11/16/18 was high in Chengdu, with HPV16 exhibiting the highest infection rate and HPV16 single infections showing an annual increase. The prevalence of HPV6/11/16/18 infection in young women (< 20 years) was highest and in old women (≥ 60 years) was higher; therefore, prioritizing vaccination for young females is essential in the current context, while HPV screening remains an effective approach for older females. Among the available HPV vaccines in Chengdu, the nonavalent vaccine appears to be relatively most suitable for Chengdu females. Additionally, in patients with genital warts, it is necessary to assess the presence of HR-HPV infection and manage it appropriately.

## Data Availability

The datasets used and/or analysed during the current study are available from the corresponding author J.Y.Z upon reasonable request.
